# Comparative Assessment of Knowledge, Attitudes, and Practice of Breast Self-Examination among Female Secondary and Tertiary School Students in Ghana

**DOI:** 10.1155/2018/7502047

**Published:** 2018-07-30

**Authors:** Linda Ahenkorah Fondjo, Osei Owusu-Afriyie, Samuel Asamoah Sakyi, Akua Addo Wiafe, Bright Amankwaa, Emmanuel Acheampong, Richard K. D. Ephraim, William K. B. A. Owiredu

**Affiliations:** ^1^Department of Molecular Medicine, School of Medical Sciences, College of Health Sciences, Kwame Nkrumah University of Science and Technology, Kumasi, Ghana; ^2^Department of Pathology, Police Hospital, Accra, Ghana; ^3^Department of Medical Laboratory Technology, Kwame Nkrumah University of Science and Technology, Kumasi, Ghana; ^4^Department of Medical Laboratory Technology, University of Cape-Coast, Cape-Coast, Ghana

## Abstract

**Background:**

Breast cancer remains a serious public health problem globally. It is particularly increasing among adolescents and premenopausal women. Breast self-examination (BSE) is the most effective and feasible means of detecting breast cancer early in developing countries. This study aimed at evaluating and comparing knowledge of BSE among secondary and tertiary school students and at revealing their attitudes and practices about BSE.

**Method:**

This cross-sectional study was conducted among 1036 female secondary and tertiary school students of Kwame Nkrumah University of Science and Technology and Technology Senior High School. Data was obtained using a pretested questionnaire to access sociodemography, knowledge, attitudes, and practice of BSE among the students.

**Result:**

Most students were within the age of 15–24 years; 90.9% were aware of BSE. A high level of knowledge on BSE was found in 54.5% of the students. Knowledge was significantly higher in tertiary than secondary school students* (p=0.002).* 24.1% of the students thought BSE could be performed anytime; however only 8.1% of the students performed BSE monthly as recommended, whilst 41.8% had never practiced. Of these, more secondary students had never practiced BSE as compared to the tertiary students. 22.3% indicated they would wait for a change in a detected breast lump before seeking medical attention. 96.3% of the participants agree BSE is a good practice which must be encouraged.

**Conclusion:**

Teaching of BSE should be intensified beginning at the high school level, emphasizing practice and its benefits for early detection of breast cancer.

## 1. Background

Cancer of the breast is a serious disease and the most common malignancy affecting women worldwide. There are reports of increased incidence with mortality rates of over 75% in developing countries where, previously, low incidences have been reported [1]. In Ghana, currently, apart from being the commonest cancer type [2], the age at diagnosis can be as early as 14 years [3] with increasing incidence pattern in premenopausal women [4]. Several previous reports in Ghana and elsewhere indicate that the average age of breast cancer incidence is between 40 and 50 years. [3–6]. Early detection of breast cancer is crucial and the most effective way of diagnosing breast cancer to reduce its morbidity and mortality [7, 8]. The three screening methods recommended by the American Cancer Society are mammography, clinical breast examination, and BSE. Mammography is the best method for early detection of breast cancer [7, 8]; however, in most developing countries, mammography is expensive and inaccessible; thus this makes BSE comparatively cheaper and easy to perform [8, 9].

BSE allows women to become aware of their breasts so as to detect changes early [9, 10]. It is simple and cost-effective in poor resource settings and in areas with limited access to health care [11]. It is common knowledge that, in Ghana, most women diagnosed with breast cancer are usually in the advanced stage of the disease. With low availability of advanced laboratory equipment like mammography for screening and diagnosis of breast cancer in Ghana coupled with the cost, as well as the current early age at diagnosis, this study sought to comparatively assess knowledge of BSE among female senior high school (SHS) students and female university students as well as to reveal their attitudes and practice about BSE. This is imperative if breast cancer is to be detected early in resource limited countries like Ghana.

## 2. Materials and Methods

### 2.1. Study Design

This descriptive cross-sectional study was conducted at the Kwame Nkrumah University of Science and Technology (KNUST) from January to May 2017 to evaluate the knowledge, attitudes, and practice of BSE among female SHS and tertiary students of the university.

### 2.2. Study Area and Population

The study was conducted at the Technology SHS and KNUST; both schools are located in Kumasi, the capital of the Ashanti region, Ghana. The university is a Science and Technology Institution, so it attracts Ghanaians students of different cultures and backgrounds and International students. The Technology Senior High School is the University's Secondary School and is comprised of mainly Ghanaian students.

## 3. Ethical Considerations

This study was approved by the Committee on Human Research, Publications and Ethics of the School of Medical Sciences, KNUST (CHRPE/AP/086/17). Approval to conduct the study at the SHS was also obtained from the school authorities, and written informed consent was obtained from all respondents after the aim of the study had been explained. All information obtained was treated as confidential.

## 4. Data Collection

A proportionate stratified sampling technique was used to recruit a total of 1036 female students comprising 359 SHS students and 677 university female students. Data for the study was obtained using a questionnaire. The questionnaire was pretested on 20 students from neighboring schools and the necessary amendments were made before administering them to the study participants. The respondents were briefed about the aims of the study before the administration of the questionnaire.

The questionnaire used consisted of three parts: sociodemographic characteristics, knowledge on BSE, and attitudes towards BSE and practice of BSE. There were 8 questions on sociodemographics, 21 questions on knowledge, and 15 questions on attitudes and practice of BSE.

The questionnaire was self-administered to participants to limit bias and was distributed to participants in their classrooms and places of residence.

## 5. Statistical Analysis

The data collected was entered into Microsoft Excel and analyzed using SPSS version 22 and GraphPad Prism version 6. Simple descriptive statistics such as frequencies and percentages were used to present the data; chi-square or Fisher's exact tests were used to test associations where appropriate. A statistical significant level was set at p< 0.05 for all tests.

## 6. Results

### 6.1. Sociodemographics of Study Participants

All one thousand and thirty-six female students (1036) recruited for the study completed and returned the questionnaires. Three hundred and fifty-nine (359) were secondary students and six hundred and seventy-seven (677) were university students. The ages of the students ranged from 15 to 35 years; majority of the SHS students (73.2%) were in the 15-19 age group, whilst most (96.4%) of the tertiary students were within the 20-24 years; 15.4% and 0.8% of participants were in the 25-30 and 30-35 age groups, respectively. The difference in age groups across educational level was statistically significant (p< 0.0001). Majority (88.2%) of the students lived in urban areas whereas 11.8% lived in rural areas. A statistically significant difference between resident types was observed among participants (p= 0.0005). Almost all participants (95.8%) were Christians, 4.0% were Muslims, and 0.2% were of other religions. Most of the students (94.8%) had no family history of breast cancer whereas 5.2% had a family history of breast cancer (Table 1).

### 6.2. Awareness and Knowledge on BSE

Almost all (90.9%) of the study participants were aware of BSE. Of these, 33.0% were SHS students and 67.0% were university students. There was statistical significant difference in awareness between the 2 groups of students (p=0.0006). 91.6% of students were able to identify BSE as tool for early detection of breast cancer. With regard to where to examine when performing a BSE, almost all (95.7%) of the students knew that the breasts were to be examined whilst 63.1% knew that the area between the breast and the collarbone was to be examined, and only 43.2% knew that the armpit was also to be examined. The knowledge on where to examine was significantly higher in university students than in the secondary school students (p <0.05) (Table 2). Less than half (45.8%) of the students knew that BSE was to be performed monthly, and 24.1%, 18.0%, and 7.3% thought it could be performed anytime, weekly, and yearly, respectively. These results were significantly higher in the university students than in the secondary school students (p <0.0001). Only 21.1% of students knew that BSE was to be performed after menstruation (66 SHS students and 153 university students). Moreover, more SHS (61.5%) than university students (38.5%) thought BSE could be performed during menstruation. There was statistical significant difference between the groups (p= 0.03) (Table 2).

More university than SHS students had adequate knowledge, on postures to assume when performing a BSE 63.0% and 41.1% of the students identified lying down and standing as postures assumed when performing a BSE, respectively. Additionally, more than half (58.2%) of the students knew a mirror was required when performing a BSE. However, only 19.0% of the students knew that the middle portion of the fingers was to be used to examine the breasts. Most students (87.6%) knew that the hands were raised during the BSE procedure, with majority of them being university students, and this knowledge was statistically significant (p = 0.038) (Table 2).

Majority (74.4%) of the students knew that the hand was to be moved in a clockwise direction when performing a BSE, and, of these, the number of university students was significantly higher than secondary school students (p=0.047). Less than half of the students (42.3%) knew that a constant pressure must be applied when performing a BSE. The number of university students who knew this was significantly higher than the SHS students (p <0.0001). In general, 54.5% had a higher level of knowledge whilst 45.5% had a low level of knowledge. University students had a significantly higher knowledge in BSE than SHS students (p=0.002) (Table 2).

### 6.3. Knowledge on Breast Cancer Symptoms

Majority of the students were able to indicate that a lump in the breast, discoloration, nipple discharge, sores, change in symmetry, and change in size of the breast were symptoms of breast cancer. However, the knowledge of breast cancer symptoms was higher in tertiary school students; this difference was statistically significant in relation to identifying a lump in the breast (p <0.0001) and nipple discharge (p=0.007) as symptoms of the disease (Table 3).

### 6.4. Attitudes towards BSE

Majority (96.3%) of participants agreed BSE was a good practice and should be encouraged. Most (82.1%) of the students stated they would report a lump they detect in their breasts to a doctor immediately. Furthermore, 6.3% of the students, however, thought BSE was time-consuming, whilst 10.3% thought BSE was not necessary since they had no family history of breast cancer. 22.3% stated that they would wait for some time to see if a lump in their breast changes before seeking medical attention. The difference in attitude between SHS and tertiary school students was statistically significant (p <0.05), except for reporting a lump in their breasts to a doctor (p=0.158). Almost all (97.1%) of the students had a good attitude towards BSE; nonetheless university students had significantly better attitudes than the secondary school students* (p =0.005)* (Table 4).

### 6.5. BSE Practices

When practice of BSE was assessed, only 8.1% of the students performed BSE monthly as recommended. 48.1% practiced BSE only once in a while, and 2.0% practiced BSE once a year, whereas 41.8% had never practiced BSE. The tertiary school students who performed BSE once a month were significantly higher than those of the secondary school, and the number of secondary school students who had never performed a BSE was significantly higher than students in the university (p <0.0001).

The foremost reason most participants performed BSE was because they were completely aware of the benefits (76.3%); subsequently, 63.9% thought they would not like to be diagnosed of breast cancer. 9.7% indicated they would perform BSE because of a family history of breast cancer or because participants knew someone with breast cancer. The difference in reasons for performing BSE was statistically significant between the 2 groups, with more secondary school than tertiary school students performing BSE because they knew someone who had breast cancer (p =0.002) (Table 5).

While some of the students (36.8%) indicated that their noncompliance to BSE was because of not knowing the right procedure, 26.7% attributed their lack of practice to forgetfulness and 12.3%, 10.5%, and 7.1% indicated not being at risk of breast cancer, fear of being diagnosed of breast cancer, and the discomfort in touching their bodies, respectively, as reasons for not performing BSE. These reasons were significantly higher in the university students than in the SHS students (p <0.05). More than half (52.3%) of the students were influenced by the media to perform BSE, followed by health workers (34.0%), and the least sources of influence were a personal history of breast cancer (1.6%) and a family history of breast cancer (2.4%). Influences from family and peers were 9.5% and 6.6%, respectively (Table 5).

**Figure 1:**
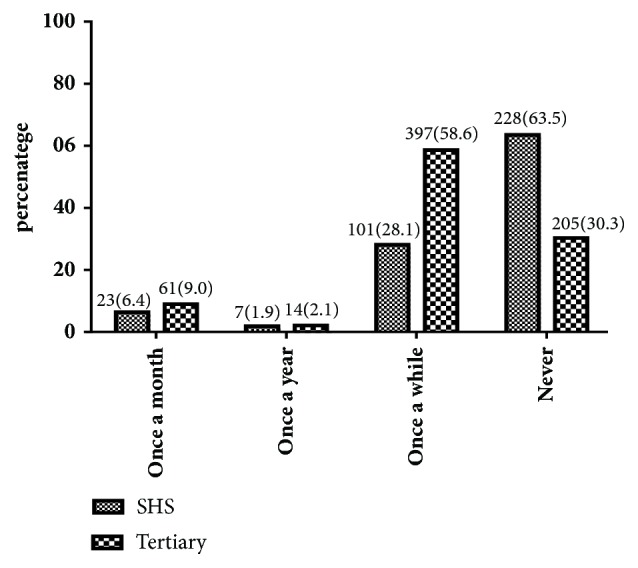
Frequency of BSE performance among study participants.

Majority of the tertiary students (58.6%) perform BSE once in a while, while most (63.5%) of the SHS students have never practiced BSE. Smallest proportions (1.9% and 2.1%) of the SHS and tertiary students, respectively, practiced BSE once a year. Furthermore, 23 and 61, accounting for 6.4% and 9.0% of the SHS students and tertiary, respectively, practiced BSE once a month (Figure 1).

## 7. Discussion

Early detection of breast cancer has by far been the ideal way to reduce breast cancer mortality worldwide. BSE has been recommended for early detection in developing countries since it is relatively a simpler and affordable method in resource limited settings. In Ghana, breast cancer continues to cause devastating outcomes due to late detection. This study assessed and compared the knowledge, among tertiary and secondary school students, and revealed the attitudes and practice of BSE among these young women. To the best of our knowledge, there has been no study that assessed and compared knowledge, attitudes, and practice in both secondary and tertiary students in one study in Ghana or elsewhere.

From this study majority of the students were aware of BSE, and most recognized it as a method for the early detection of breast cancer. This finding is similar to studies conducted in Cameroon and Uganda [12, 13] but on the contrary to Turkish study conducted among high school students where majority of the students were unaware of BSE [14]. The level of awareness was however higher in the university students than in SHS students from our study (Table 2).

In the present study, barely more than half of participants had a high knowledge on BSE. This finding is relatively lower compared to a study among nursing students in India where 84.0% had a high knowledge on BSE [15]. This could be as a result of the fact that the nursing students are taught BSE as part of their academic curriculum. Though the general BSE knowledge level was above average, most SHS students had a low level of knowledge on BSE (Table 2). Educational level was found to be significantly associated with good knowledge and practice of BSE, a finding consistent with reports from Balogun and Owoaje [16] and Ayed et al. [7] (Table 2).

The percentage of students aware that BSE was to be performed monthly from our study was 45.8%, with 21.1% knowing that it was to be performed after menstruation. Similar findings were reported by Suh et al. [12] and Karayut et al. [14]. This knowledge and awareness was significantly lower in secondary school students than in university students, as only 39.2% and 30.1% of the students knew the right frequency and time to perform BSE (Table 3).

The observation that 97.1% had good attitudes towards BSE from this study is consistent with a study among medical students in Saudi Arabia [17]. However, majority with poor attitudes were found to be SHS students (Table 4). A good attitude observed among the students was that majority stated they would report a lump in their breasts immediately to a doctor, a finding comparable to an earlier study among the students of Presbyterian University College, in Ghana [18]. 96.3% of the students acknowledged the necessity for BSE and agreed it should be encouraged, a finding similar to a Ghanaian study among tertiary students by Sarfo et al. [18]. Except that, a higher percentage was observed in our present study; this could be attributed to the larger sample size used in our study.

More than half of the SHS students stated that BSE was time-consuming and was not necessary especially since they had no family history of breast cancer (Table 4), an erroneous belief that needs to be addressed. Moreover, in a recent descriptive cross-sectional study among female breast cancer patients in Ghana by Fondjo et al. [5], most of the women did not have a family history of breast cancer prior to their diagnosis. This indeed calls for a deepened education on the benefits of BSE especially among the secondary school students if breast self-examination is to be inculcated at an early age for early detection and prevention.

From this present study, in line with the recommended once a month frequency, only 8.1% practiced the examination (Table 5). This finding is comparable to studies conducted in Nigeria, Saudi Arabia, and Ghana [17–19], where low practice has also been reported. However, our finding is in contrast with the findings from a study among Ghanaian market women where 64.0% were reported to practice BSE once a month [20]. This could be due to the fact that most market women are older and are much more aware that being postmenopausal is a risk factor for breast cancer and would want to detect the condition early.

A huge number of the study participants had never performed a BSE, with majority of those who never performed being SHS students. An observation attributed to their lack of knowledge on the right procedure for the examination, a finding similar to reports from other comparable studies [9, 14, 20–22]. This study is limited by the fact that there was no question asking if students have received training on how to perform BSE, except for nursing students who are trained as part of their academic and professional practice. However, this does not have effect on the aims and findings of this study but contributes to scientific knowledge.

The focus group for awareness campaigns should be the senior high schools and breast screening started preferably from the early 20s [4] due to the changing incidence trends in Ghanaian women. The results of this study indeed strengthen the need for planning strategies to deepen awareness on BSE as a screening method for early detection of breast cancer.

## 8. Conclusion

The practice of BSE in line with the recommended once a month frequency was extremely low in both the secondary and tertiary students, with the secondary school students having a poorer knowledge on BSE. BSE training must be incorporated into second cycle education. Awareness campaigns and training must be intensified, with more focus on the adolescent, beginning at the SHS level and emphasizing its practice.

## Figures and Tables

**Table 1:** Sociodemographics of study participants stratified by their educational level.

**Variable**	**Total **	**SHS **	**Tertiary **	**x** ^**2**^,	***P-value***
	**n**(%)	**n**(%)	**n**(%)	**df**	
**Age (years)**					**<0.0001**
15-19	463(44.7)	339(73.2)	124(26.8)		
20-24	549(53.0)	20(3.6)	529(96.4)		
25-30	16(15.4)	0(0.0)	16(100.0)		
31-35	8(0.8)	0(0.0)	8(100.0)		
**Residence**					**0.0005**
Rural	122(11.8)	60(53.6)	62(55.4)		
Urban	914(88.2)	299(32.7)	615(67.3)		
**Religion**				1.23, 2	0.541
Christianity	993(95.8)	342(34.4)	651(65.6)		
Islam	41(4.0)	17(41.5)	24(58.5)		
Others	2(0.2)	0(0.0)	2(100.0)		
**Family History of BC**					0.769
Yes	54(5.2)	20(37.0)	34(63.0)		
No	982(94.8)	339(34.5)	643(65.5)		

SHS= senior high school, BC= breast cancer, df= degree of freedom.

**Table 2:** Participants knowledge on BSE stratified by their educational level.

**Variable**	**Total, **	**SHS, **	**Tertiary, **	**x** ^**2**^ **, df**	**p-value**
	**n**(%)	**n**(%)	**n**(%)		
**Have you heard of BSE **					**0.0006**
Yes	942(90.9)	311(33.0)	631(67.0)		
No	94(9.1)	48(51.1)	46(48.9)		
**Is it necessary to perform BSE**					**0.011**
Yes	1006(97.1)	355(35.3)	651(64.7)		
No	30(2.9)	4(13.3)	26(86.7)		
**BSE is a practice to detect BC early **					**0.003**
Yes	949(91.6)	316(33.3)	633(66.7)		
No	87(8.4)	43(49.4)	44(50.6)		
**Areas to examine when performing BSE **					
**Breast**					0.635
Yes	991(95.7)	342(34.5)	649(65.5)		
No	45(4.3)	17(37.8)	28(62.2)		
**Armpit**					**<0.0001**
Yes	448(43.2)	120(26.8)	328(73.2)		
No	558(53.9)	239(42.8)	349(62.5)		
**Between breast and collarbone**					**<0.0001**
Yes	654(63.1)	258(39.4)	396(60.6)		
No	382(36.9)	101(26.4)	281(73.6)		
**How often must BSE be performed **				24.0,4	**<0.0001**
Weekly	186(18.0)	58(31.2)	128(68.8)		
Monthly	475(45.8)	186(39.2)	289(60.8)		
Yearly	76(7.3)	37(48.7)	39(51.3)		
Anytime	250(24.1)	60(24.0)	190(76.0)		
Don't know	49(4.7)	18(36.7)	31(63.3)		
**When is the right time to perform BSE **				11.0,4	**0.03**
Before menstruation	129(12.5)	42(32.6)	87(67.4)		
During menstruation	26(2.5)	16(61.5)	10(38.5)		
After menstruation	219(21.1)	66(30.1)	153(69.9)		
Anytime	590(56.9)	209(35.4)	381(64.6)		
Don't know	72(6.9)	26(36.1)	46(63.9)		
**Postures for BSE **					
**Standing**					0.426
Yes	426(41.1)	154(36.2)	272(63.8)		
no	610(58.9)	205(33.6)	405(66.4)		
**Lying down**					**<0.0001**
Yes	653(63.0)	268(41.0)	385(59.0)		
No	383(37.0)	91(23.8)	292(76.2)		
**Sitting**					**<0.0001**
Yes	207(20.0)	157(75.8)	50(24.2)		
No	829(80.0)	202(24.4)	627(75.6)		
**Is mirror required for BSE **					0.99
Yes	603(58.2)	210(34.8)	393(65.2)		
No	67(6.5)	23(34.3)	44(65.7)		
**Part of the hand used **				4.0,3	0.266
Finger tips	603(58.2)	206(34.2)	397(65.8)		
Middle part of fingers	197(19.0)	60(30.5)	137(69.5)		
Palm	168(16.2)	66(39.3)	102(60.7)		
Don't know	68(6.6)	27(39.7)	41(60.3)		
**In one step the hands are raised **					**0.038**
Yes	908(87.6)	304(33.5)	604(66.5)		
No	128(12.4)	55(43.0)	73(57.0)		
**Direction of hand movement during BSE **				6.0,2	**0.047**
Random	197(19.0)	82(41.6)	115(58.4)		
Clockwise	771(74.4)	251(32.6)	520(67.4)		
Don't know	68(6.6)	26(38.2)	42(61.8)		
**The pressure applied in BSE is**				56.0,3	**<0.0001**
Constant	438(42.3)	114(26.0)	324(74.0)		
Varied	150(14.5)	44(29.3)	106(70.7)		
Of constant and varying degrees	332(32.0)	168(50.6)	164(49.4)		
Don't know	116(11.2)	33(28.4)	83(71.6)		
**Level of knowledge**					**0.002**
Low	471(45.5)	187(39.7)	284(60.3)		
High	565(54.5)	172(30.4)	393(69.6)		

SHS= senior high school, BSE= breast self-examination, BC= breast cancer, df= degree of freedom.

**Table 3:** Participants' knowledge on symptoms of breast cancer stratified by their educational level.

**Knowledge of BC symptoms**	**Total **	**SHS **	**Tertiary **	**p-value**
	**n**(%)	**n**(%)	**n**(%)	
**Lump**				**<0.0001**
Yes	920(88.8)	291(31.6)	629(68.4)	
No	116(11.2)	68(58.6)	48(41.4)	
**Discoloration**				0.079
Yes	709(68.4)	233(32.9)	476(67.1)	
No	327(31.6)	126(38.5)	201(61.5)	
**Nipple discharge**				**0.007**
Yes	875(84.5)	288(32.9)	587(67.1)	
No	161(15.5)	71(44.1)	90(55.9)	
**Sores**				0.09
Yes	794(76.6)	264(33.2)	530(66.8)	
No	242(23.4)	95(39.3)	147(60.7)	
**Change in breast symmetry**				0.354
Yes	796(76.8)	282(35.4)	514(64.6)	
No	240(23.2)	77(32.1)	163(67.9)	
**Change in breast size**				0.587
Yes	799(77.1)	273(34.2)	526(65.8)	
No	237(22.9)	86(36.3)	151(63.7)	

BC: breast cancer.

**Table 4:** Participants attitudes towards BSE stratified by their educational level.

**Variables**	**Total, **	**SHS,**	**Tertiary, **	**x** ^**2**^,	**p-value**
	**n(%)**	** n(%)**	**n(%)**	**df**	
**I think BSE is time consuming **				10.0,2	**0.007**
agree	65(6.3)	34(52.3)	31(47.7)		
not so sure	194(18.7)	69(35.6)	125(64.4)		
disagree	777(75.0)	256(32.9)	521(67.1)		
**No FH of BC so no need to practice BSE **				19.9,2	**<0.0001**
agree	107(10.3)	57(53.3)	50(46.7)		
not so sure	105(10.1)	40(38.1)	65(61.9)		
disagree	824(79.5)	262(31.8)	562(68.2)		
**I will report a lump after waiting for a while**				14.5,2	**0.0007**
agree	231(22.3)	101(43.7)	130(56.3)		
not so sure	217(20.9)	81(37.3)	136(62.7)		
disagree	588(56.8)	177(30.1)	411(69.9)		
**I will immediately report a lump in my breasts**				3.7,2	0.158
agree	851(82.1)	301(35.4)	550(64.6)		
not so sure	112(10.8)	30(26.8)	82(73.2)		
disagree	73(7.0)	28(38.4)	45(61.6)		
**BSE is a good practice, and all women must be taught**				10.6,2	**0.005**
agree	998(96.3)	337(33.8)	661(66.2)		
not so sure	25(2.4)	16(64.0)	9(36.0)		
disagree	13(1.3)	6(46.2)	7(53.8)		
**Attitude score**					**0.005**
Good attitude	1006(97.1)	341(33.9)	665(66.1)		
Poor attitude	30(2.9)	18(60.0)	12(40.0)		

**Table 5:** Participants' BSE practices stratified by their level of education.

**Variables**	**Total, **	**SHS, **	**Tertiary,**	**x** ^**2**^,	**p-value**
	**n**(%)	**n**(%)	**n**(%)	**df**	
**I practice BSE because **					
**I do not want to be diagnosed BC**				6.4,2	**0.041**
agree	662(63.9)	247(37.3)	415(62.7)		
not so sure	216(20.8)	61(28.2)	155(71.8)		
disagree	158(15.3)	51(32.3)	107(67.7)		
**I am fully aware of its benefits**				6.0,2	0.05
agree	791(76.3)	277(35.0)	514(65.0)		
not so sure	205(19.8)	62(30.2)	143(69.8)		
disagree	40(3.9)	20(50.0)	20(50.0)		
**I have a family history of breast cancer/ know someone who has it**				65.5,2	**<0.0001**
agree	100(9.7)	53(53.0)	47(47.0)		
not so sure	126(12.2)	76(60.3)	50(39.7)		
disagree	810(78.2)	230(28.4)	580(71.6)		
**I do not practice BSE because**					
**I am afraid of being diagnosed of BC**				62.8,2	**<0.0001**
agree	109(10.5)	55(50.5)	54(49.5)		
not so sure	168(16.2)	65(38.7)	103(61.3)		
disagree	759(73.3)	239(31.5)	520(68.5)		
**I do not know how it is done correctly**				30.8,2	**<0.0001**
agree	381(36.8)	173(45.4)	208(54.6)		
not so sure	215(20.8)	62(28.8)	153(71.2)		
disagree	440(42.5)	124(28.2)	316(71.8)		
**I do not like touching my body**				15.6,2	**0.0004**
agree	74(7.1)	34(45.9)	40(54.1)		
not so sure	81(7.8)	41(50.6)	40(49.4)		
disagree	881(85.0)	284(32.2)	597(67.8)		
**I forget to practice BSE**				16.3,2	**0.0003**
agree	277(26.7)	77(27.8)	200(72.2)		
not so sure	181(17.5)	51(28.2)	130(71.8)		
disagree	578(55.8)	231(40.0)	347(60.0)		
**I'm not at risk of getting breast cancer**				8.9,2	**0.011**
agree	127(12.3)	59(46.5)	68(53.5)		
not so sure	120(11.6)	39(32.5)	81(67.5)		
disagree	789(76.2)	261(33.1)	528(66.9)		
**Influence to Perform BSE**				48.1	**<0.0001**
Family	72(9.5)	31(43.1)	41(56.9)		
Peers	50(6.6)	15(30.0)	35(70.0)		
Family history of Breast Cancer	18(2.4)	2(11.1)	16(88.9)		
Advice by health workers	259(34.0)	65(25.1)	194(74.9)		
The media	398(52.3)	124(31.2)	274(68.8)		
Personal history of breast cancer	17(1.6)	9(52.9)	8(47.1)		
Nothing in particular	222(21.4)	113(50.9)	109(49.1)		

## Abbreviations

BSE: Breast self-examination
SHS: Secondary high school
BC: Breast cancer.
